# Supplementary figures and images for: Long-term effects of reovirus strain T3D on the myocardium
Source: Microbiol Spectr. 2026 Jan 9;14(2):e02108-25. doi: 10.1128/spectrum.02108-25 (PMC12889040; doi:10.1128/spectrum.02108-25)

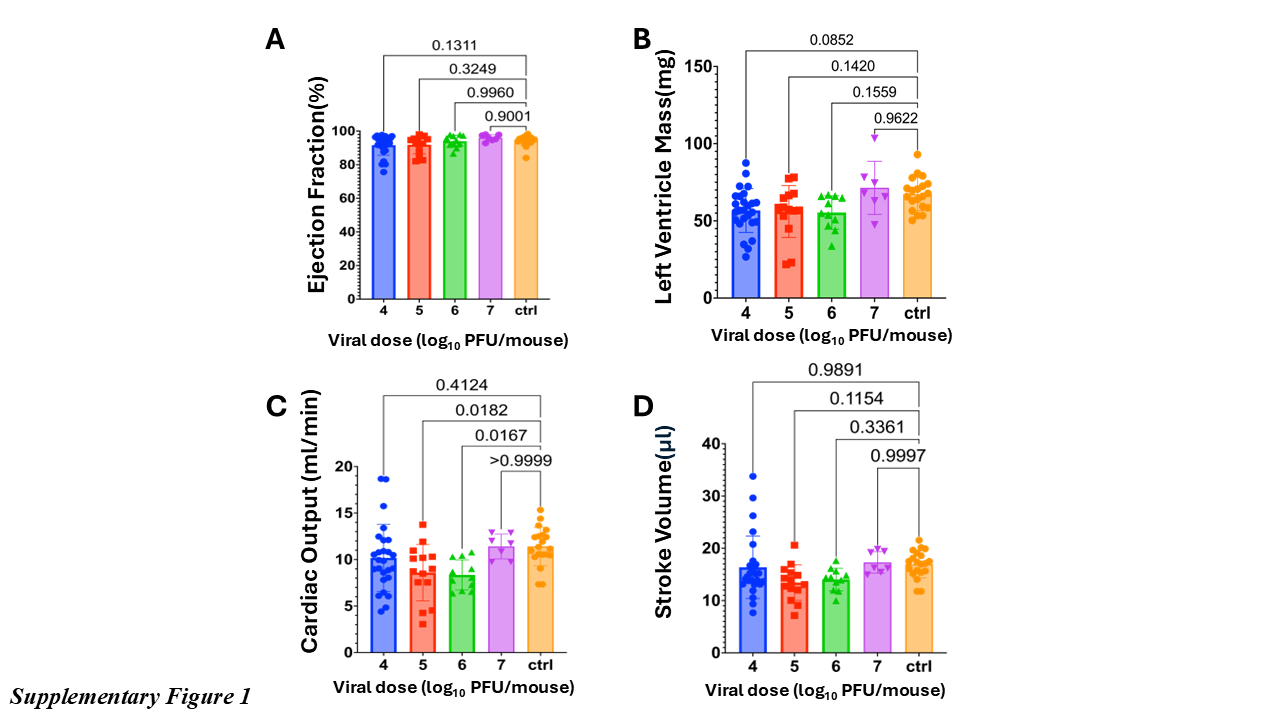

Supplement: Fig. S1 — Echocardiography analysis at 4 weeks post-infection. [file spectrum.02108-25-s0001.tif]

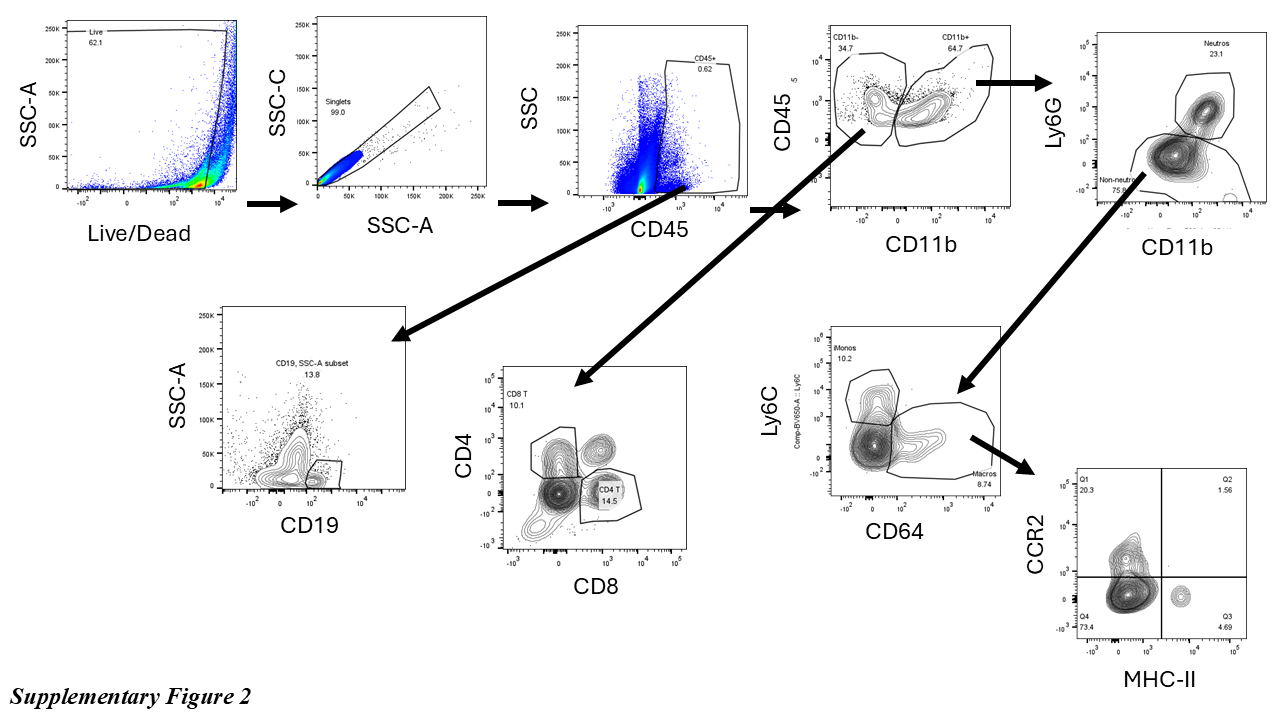

Supplement: Fig. S2 — Flow cytometry gating strategy. [file spectrum.02108-25-s0002.tif]
