# Supplementary material for: Long-term effects of reovirus strain T3D on the myocardium
Source: Microbiol Spectr. 2026 Jan 9;14(2):e02108-25. doi: 10.1128/spectrum.02108-25 (PMC12889040; doi:10.1128/spectrum.02108-25)
Supplement: Supplemental material — Supplemental figure legends. [file spectrum.02108-25-s0003.docx]

***Supplementary Figure 1.* Echocardiography analysis at** **4 weeks post-infection**. Neonatal C57BL/6 mice were mock infected (Ctrl; N = 20) or infected orally with 10^4^ (N = 26), 10^5^ (N = 14), 10^6^ (N = 11), or 10^7^ (N = 7) PFU of T3D. (A) Ejection Fraction, (B) Left Ventricular Mass, (C) Cardiac Output, and (D) Stroke Volume were measured at 26-35 days post-inoculation. *P*-values represent ANOVA followed by pairwise comparisons, with *p*-values adjusted using the False Discovery Rate method.

***Supplementary Figure 2.* Flow cytometry gating strategy**

Single-cell suspensions from digested neonatal mouse hearts (PBS- or T3D-infected) were stained and analyzed by flow cytometry. Viable cells were first identified as Zombie Aqua-negative, and singlets were selected to exclude aggregates and doublets. CD45⁺ leukocytes were then gated from total cardiac cells. Within the CD45⁺ population, CD11b⁺ myeloid and CD11b⁻ lymphoid cells were distinguished. Among the myeloid cells, Ly6G⁺CD11b⁺ cells were classified as neutrophils, while Ly6G⁻CD11b⁺ cells were further divided into Ly6C⁺CD64⁺ monocytes and Ly6C⁻CD64⁺ macrophages. Macrophage subsets were subsequently characterized based on MHC-II and CCR2 expression to identify CCR2⁺MHC-II⁺ inflammatory and CCR2⁻MHC-II⁺ resident populations. Within the lymphoid compartment, CD19⁺ cells represented B cells, and CD4⁺ and CD8⁺ subsets were identified as helper and cytotoxic T cells, respectively. Percentages indicate the proportion of the gated population relative to its parental gate. Representative plots were generated in FlowJo from cardiac single-cell suspensions analyzed 26 days post-infection.
